# Efficacy of a parent-based treatment for children and adolescents with obsessive-compulsive disorder: Protocol of a multiple baseline, single-case experimental design study

**DOI:** 10.1016/j.conctc.2025.101456

**Published:** 2025-02-11

**Authors:** Julia D.K. Veeger, Luuk Stapersma, Eli R. Lebowitz, Bonne Zijlstra, Ramón Lindauer, Elisabeth M.W.J. Utens, Chaim Huijser

**Affiliations:** a Amsterdam UMC, Location University of Amsterdam, Department of Child and Adolescent Psychiatry, Amsterdam, the Netherlands; b Academic Center for Child and Adolescent Psychiatry Levvel. Amsterdam, the Netherlands; c Research Institute of Child Development and Education, University of Amsterdam, Amsterdam, the Netherlands; d Yale University Child Study Center, New Haven, CT, USA

**Keywords:** Obsessive-compulsive disorder, Children, Adolescents, Parent-based treatment, Family accommodation, SPACE

## Abstract

**Background:**

Pediatric obsessive-compulsive disorder (OCD) is a severely impairing disorder, associated with high levels of family accommodation (FA). Approximately 40 % of youth do not benefit from first-line treatment options (cognitive behavioral therapy or pharmacotherapy). Supportive Parenting for Anxious Childhood Emotions (SPACE) is a parent-based treatment, teaching parents to reduce FA and increase supportive parenting, thereby aiming to improve the child's OCD. This article presents the protocol of a multiple baseline single-case experimental design (SCED) study to test the efficacy of SPACE in reducing OCD severity and FA in youth with OCD.

**Methods:**

This SCED consists of a baseline, treatment, and follow-up phase. In total 25 youth (7–18 years) with OCD, who previously received cognitive behavioral therapy (CBT) unsuccessfully, aborted treatment early, or were not able to receive CBT due to too high levels of OCD/anxiety, and their parents will be included. They will be randomly allocated to one of three baseline phase options (4, 6 or 8 weeks). The treatment phase consists of 12 weekly sessions of SPACE with parents. Throughout all phases, OCD severity and FA will be briefly assessed thrice a week. Standard clinical measurements assessing OCD severity and FA and secondary parameters will be conducted at six timepoints, till 6 months follow-up.

**Conclusion:**

Combining the innovative SPACE treatment with a SCED provides detailed insight into the relationship between OCD and FA over time. Studying this in clinical practice in complex cases that are normally understudied, helps to improve more personalized care for youth with OCD.

## Highlights

• Almost 40 % of youth with obsessive-compulsive disorder do not benefit from first line treatment.
• SPACE is a parent-based treatment.
• Parents learn to support the child and reduce family accommodations.
• SPACE is found to be as effective as CBT in youth with anxiety disorders.
• This study presents the first SCED protocol to examine the efficacy of SPACE for pediatric obsessive-compulsive disorder.

## 1 Background

Children and adolescents with obsessive-compulsive disorder (OCD) experience recurrent obsessions and compulsions causing significant impairments in their daily activities and family functioning. Obsessions are anxious or distress-provoking intrusive thoughts, images, or impulses. Compulsions are time-consuming ritualistic behaviors (e.g., repetitive checking, cleaning) or mental tasks (so-called cognitive compulsions, e.g., repeating sentences in one's head). OCD affects 0.5–2% of children [1,2] and is characterized by late recognition of symptoms, delayed start of treatment, and, if not treated, a chronic and disabling disease course that can disrupt development during childhood and adolescence [3].

Evidence-based first-line treatment options for pediatric OCD are cognitive behavioral therapy (CBT, including exposure and response prevention (ERP)) and pharmacotherapy (especially selective serotonin reuptake inhibitors; SSRI's). However, 40 % of children with OCD do not respond to these treatments [4,5] or drop out of treatment prematurely. In clinical trials 10 % of youth with OCD drop out of ERP treatment and 17 % drop out of pharmacotherapy [6]. In addition, little is known about treatment refusal rates in children with OCD, but it is common in clinical practice due to low motivation or high anxiety rates. Additional alternative treatment options for these unreached and often complex OCD cases are therefore urgently needed.

Increasing attention is being given to family factors that play a role in childhood anxiety and OCD [7,8], in particular to family accommodation (FA) [9,10]. FA encompasses behaviors of parents, siblings, and other caregivers to alleviate distress in the affected child. In families of children with OCD and other anxiety problems, FA is highly prevalent and significantly elevated compared with families of children without clinical anxiety disorders [11,12]. Higher FA is associated with greater severity of symptoms and poorer treatment outcomes in children and adolescents [13].

To improve treatment outcomes for children with OCD and anxiety, Lebowitz and colleagues [14] developed an innovative parent-based (stand-alone) treatment targeting FA in relation to children's anxiety and/or OCD: *Supportive Parenting for Anxious Childhood Emotions* (SPACE). A unique advantage of the program is that SPACE is a completely parent-based treatment protocol that can be applied without cooperation of the child, and thus can be used for families of children who are reluctant or not motivated to participate in treatment or do not respond sufficiently to CBT. Parents are taught to reduce FA and to increase supportive responses towards their child.

The theoretical foundation of SPACE is based on several perspectives, such as attachment theory [15], systemic therapy, CBT and Non-Violent Resistance (NVR) [16]. Attachment theory describes the mutually interactive bond between parents and children, in which parents are inclined to protect and respond to fear or distress cues of the child and the child largely depends on the parent in dangerous or stressful situations [15]. Anxiety activates this bond; the child seeks protection from the perceived danger and the parent provides protection and regulation. Within the SPACE framework, therefore, anxiety is considered as an interpersonal and systemic construct. Protective responses of parents to their child's OCD and anxiety can be considered as repeatedly activating the attachment system. Children with OCD and/or anxiety often have difficulties with self-regulating their emotions, which can be maintained by the protective responses of parents [17]. Then, within the family, a kind of maladaptive equilibrium may arise, in which the child relies on the repeated reassuring accommodation of parents. NVR provides a framework for altering this maladaptive equilibrium. The core principle of NVR is that in situations of conflict, productively acting relies on changing one's own behavior, and not focusing on changing the other's behavior. This can help parents to avoid being drawn into the child's demanding or coercive disruptive behavior dynamics that provoke family accommodations [18,19].

Empirical studies, primarily in the United States (US) have made clear that SPACE is promising. SPACE was evaluated in a non-inferiority randomized controlled trial (RCT), and found to be as efficacious as CBT in decreasing childhood anxiety and significantly more efficacious in decreasing FA [20]. More recently, both a low-intensity version of SPACE and the standard SPACE treatment protocol showed strong treatment effects for anxious youth, including OCD [21]. A case series showed that SPACE is a feasible and acceptable treatment option for parents of children with OCD [14]. However, SPACE has not been evaluated as rigorously outside the US and in youth with OCD. The current study aims to investigate the efficacy of SPACE on OCD severity and FA in Dutch youth with complex OCD, with a multiple baseline single-case experimental design (SCED). Secondly, we aim to identify predictors and moderators of treatment response (such as comorbidity, parental burden, and parental psychopathological symptoms) and gain more insight into the mechanisms of change. A major advantage of exploring an innovative parent-based treatment with a SCED is that this allows to gain more detailed insights into changes and barriers in the treatment over time in complex cases of youth with OCD.

## 2 Design and methods

### 2.1 Study design

This study uses a multiple baseline single-case experimental design (SCED) to examine the efficacy of the SPACE program in 25 children and adolescents and their families. A SCED uses a within-subject design to monitor in detail treatment effects on a case-by-case basis over time [22]. It is characterized by frequent repeated assessments of the individual's behavior across multiple phases with the intervention being absent and present. SCED is specifically suitable for innovative interventions for relatively small populations (such as youth with complex OCD) [[22], [23], [24]]. Importantly, using detailed, repeatedly measured data of participants, SCED's have sufficient statistical rigor comparable to larger group-based designs. With these characteristics, SCED's help to bridge the gap between science and practice [[25], [26], [27]].

The course of this study follows a multiple baseline design with three phases: the baseline phase (A, no treatment), treatment phase (B, treatment) and follow-up phase (C, no treatment). To assess primary outcomes, participants complete frequent brief assessments on OCD severity and FA thrice a week throughout all phases, resulting in 72 time points assessing OCD and FA, for SCED analyses. The treatment phase will be split up in two distinct phases: B1 and B2. The division in phases is made at the time parents implement their first plan to reduce a target accommodation (see *2.5. Intervention)*. We aim to explore whether the moment of implementation (start of phase B2) brings a major change in accommodation behavior and whether the actual implementation can be seen as a core working mechanism of SPACE. B1 includes the SPACE sessions before parents implement their plan and are dedicated to psychoeducation, increasing supportive responses, charting accommodations, choosing target behaviors, and formulating a plan. Implementation of the plan is not fixed to a certain treatment session, so the transition between B1 and B2 can alternate between participants, indicating the suitability of the SCED approach. It has been shown that including six participants in a SCED design is considered satisfactory to observe moderate treatment effects [28]. At least nine case series demonstrating efficacy are needed to consider an intervention as well established [29]. Based on previous studies investigating SPACE in children with anxiety and OCD, we expect 16–25 % to drop out of the SPACE treatment [20,30]. Therefore, this study aims to provide the SPACE intervention to 25 families and analyze all data conform the A-B1-B2-C design.

**Fig. 1:**
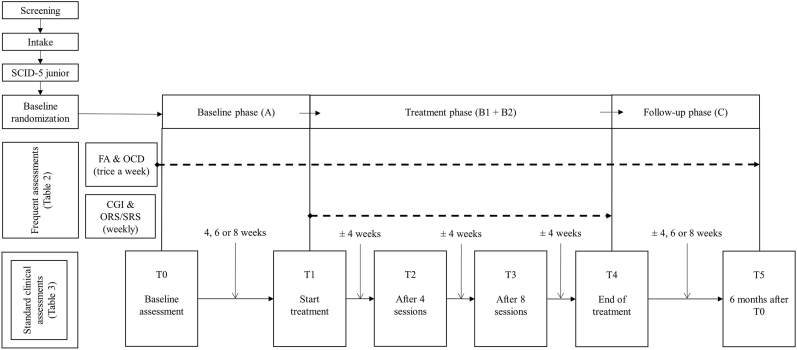
Overview patient procedures and randomization SCED.

Additionally to the SCED design, comprehensive standard clinical assessments are carried out to assess for pre-, during-, post- and follow-up differences at the following six timepoints: T0: baseline assessment during intake; T1: at the end of the baseline phase (four, six or eight weeks after T0), before treatment starts; T2: after four treatment sessions (on average four weeks after T1); T3: after eight treatment sessions (on average eight weeks after T1); T4: after all 12 treatment sessions are completed (on average 12 weeks after T1); T5: follow-up assessment, six months after T0. Moreover, every treatment session parents report about their well-being and working alliance and the therapist report about illness severity and improvement. An overview of all assessments is provided in Fig. 1 and Table 1.

**Table 1:** Core components and additional modules of the SPACE treatment.

	Component	Content
	*Core component sequence*	
1	Introduction	Setting the stage. Parents are introduced to the main aim of the SPACE program and receive psychoeducation about anxiety and/or OCD and their involvement in their child's behavior.
2	Increasing supportive responses	Parents explore how they tend to respond to the child's anxiety/OCD, are taught the concept of supportive responses and practice supportive statements.
3	Charting Accommodation	Parents learn to recognize their own accommodation behaviors and are asked to chart their accommodations in a dairy.
4	Choosing Target	Parents choose an accommodation behavior they want to reduce and set this target as a treatment goal.
5	Formulating Plan	Parents work with the therapist on a detailed plan how to implement a change in their target accommodation. Parents learn how they can inform their child, making a so-called announcement in the form of a letter to their child.
6	Implementation	Parents implement their plan and discuss progress with the therapist during the sessions.
7	Choosing Additional Target	Parents choose a second accommodation behavior to reduce and discuss the plan with the therapist.
8	Implementation of second target	Parents implement their plan to reduce the second target and discuss progress with the therapist during the sessions.
9	Summery and Termination	Parents review the progress this with the therapist. Any unclarities are resolved and the therapist discusses how parents can sustain their behavioral change.

	*Additional modules*	
1	Teaching and modelling self-regulation	Parents learn self-regulation skills to better cope with the child's distress. For example, relaxing breathing, muscle relaxation, cognitive restructuring, and self-talk.
2	Coping with disruptive behavior	Equip parents with tools to cope with disruptive or aggressive episodes of the child with NVR techniques (e.g., teaching parents to delay their response, utilize supporters, convey the severity of the child's behavior without being accusatory.
3	Coping with treats to self	Parents learn to respond appropriately when a child express treats to self by not ignoring the threat and not allowing the threat to undermine their determination to overcome the child's OCD.
4	Accessing support	Encouragement to enlist supporters from outside to bolster the parents and child in dealing with difficult processes.
5	Improving collaboration between parents	Integration of both parents' point of view and emphasizing supporting the child requires both empathic acceptance and reducing accommodation. In this module exercises as role play are used.

### 2.2 Participants

***Inclusion:*** Families are eligible to participate in this study if they meet all of the following criteria:

(a) the child is referred to Levvel, Academic Center for Child and Adolescent Psychiatry, in Amsterdam between November 2023 and December 2024 for OCD treatment;
(b) the child is between the age of 7–18 years old;
(c) at time of baseline the child still meets the clinical cut-off of 16 or higher for OCD on the Child Yale-Brown Obsessive-Compulsive Scale (CY-BOCS) [31] and the child meets the DSM-5 criteria for OCD (as primary classification) as assessed with the Structured Clinical Interview for DSM-5 disorders (SCID-5 Junior);
(d) psychiatric comorbidities of the child are included, provided that OCD is the primary treatment target;
(e) parents report high levels of family accommodation (FA) at time of baseline (meeting the cut-off of 10 or higher on the Family Accommodation Scale Anxiety (FASA) [11]);
(f) the child did not benefit from previous psychological treatment for OCD (he/she either followed 8 or more sessions of CBT or other psychotherapy), dropped out from treatment early or was unable/not motivated to receive individual treatment due to too high levels of anxiety or OCD;
(g) children are allowed to use medication or selective serotonin reuptake inhibitors (SSRI's) for OCD symptoms, provided that the medication regime has been stable 4 weeks prior to participation and is expected to remain unchanged during participation.

***Exclusion:*** families meeting any of the following criteria will be excluded:

(a) the child needs inpatient treatment;
(b) parents are not willing to participate;
(c) the child displays acute suicidality;
(d) the child displays psychotic symptoms;
(e) parents have insufficient mastery of the Dutch language;
(f) parents or child have an estimated IQ below 75. IQ will not be tested during screening for this study, but a clinical judgement is made by the clinician during screening or intake, based on school career and medical file of the child.

### 2.3 Patient recruitment and procedures

Children and their parents, referred to our academic center of expertise for youth with OCD and meeting the inclusion criteria, are contacted, and briefly informed about the research project by telephone by the researcher. During this call, the researcher (JV) conducts a first short screening to verify whether the family and the child are suitable for this research program. When a family seems eligible, a regular intake interview is planned with a clinician. After this intake interview, in which the inclusion- and exclusion criteria are checked in detail, eligible parents and children will be orally informed about the SCED study design by the researcher. Parents and children will also receive written information about the study procedures. Written informed consent will be obtained from all parents/guardians, children aged 12 and above and the teacher of the child. Only parents and children (≥12 years) who have given informed consent, will participate in this study. After informed consent has been obtained, a structured clinical interview for DSM-5 disorders (SCID-5 Junior) will be conducted with parents and children to substantiate the primary OCD classification and assess psychiatric comorbidities [32].

All participants then follow the structure of the three SCED phases: a baseline phase (A) of either four, six or eight weeks, a treatment phase (B1 and B2) consisting of 12-sessions SPACE during a period of approximately 12 weeks and a follow-up phase (C), which lasts until 6 months after the start of the baseline phase. Outcome measures are assessed with frequent brief assessments and standard clinical assessments. The majority of the instruments used in this study is part of standard clinical care and consists of psychometrically validated and sound instruments. An overview of participants procedures and randomization is provided in Fig. 1.

### 2.4 Randomization

Participants will be randomly allocated to a baseline length of four, six or eight weeks, to ensure internal validity [33]. Permuted block randomization will be used with blocks of 3 and 6 and be computerized (by the data collection system Castor EDC). It is not possible to blind the researcher conducting the assessments, since the following assessments (T1 – T5, see Fig. 1) depend on the baseline period that is randomly selected. All questionnaires will be completed online to reduce researcher influence.

### 2.5 Intervention

SPACE is a *parent-based treatment*, which means that the child will not be in contact with the therapist. The current study will follow the published SPACE protocol by the original developers, using an authorized Dutch translation [14,34]. SPACE consists of 12 weekly 60-min sessions. The first sessions focus on discussing the problems of the child and the rationale for the SPACE program. In later sessions, parents are taught supportive responses. Supportive responses include both acknowledgement of the child's anxious feelings as well as expressing confidence in the child's abilities to cope with these feelings. FA is then extensively explored, and a target accommodation is chosen to be modified as a first step. Modification of the parent's behavior follows a detailed plan. Parents implement the plan by informing the child about the plan with an announcement in the form of a letter to their child. Next, treatment focusses on persisting in following the plan. When the accommodation is successfully reduced, another target is chosen. The SPACE program also includes additional modules for teaching parents to deal with child's reactions to the FA reducing behavioral changes of the parent. The core component sequence and additional modules of the SPACE treatment are shown in Table 2.

**Table 2:** Overview of the primary and secondary outcomes measures and assessment moments.

Instrument	Variable	Assessment moment and informant					
*Primary outcome measure*		Trice every week between T0 and T5 (72 assessments)					
Frequent assessment on OCD and FA	OCD and FA severity and time	P/C					

*Secondary outcome measures*		T0	T1	T2	T3	T4	T5
Children's Yale-Brown Obsessive-Compulsive Scale (CY-BOCS)^a^	OCD Symptoms	P/C					
Children's Yale-Brown Obsessive-Compulsive Scale (CY-BOCS)^a^	OCD severity	P/C	P/C	P/C	P/C	P/C	P/C
Family Accommodation Scale- Anxiety (FASA)	Family Accommodation	P	P	P	P	P	P
Family Accommodation Scale- Anxiety – Child Report (FASA-CR)	Family Accommodation	C	C	C	C	C	C
Coercive Disruptive Behavior Scale (CD-POC)	Coercive disruptive behavior child	P	P	P	P	P	P
Child Behavior Checklist (CBCL)	Emotional and behavioral problems child	P				P	P
Teacher report form (TRF)	Emotional and behavioral problems child	T				T	T
Youth Self Report (YSR)	Emotional and behavioral problems child	C				C	C
Screen for Childhood Anxiety Related Emotional Disorders (SCARED)	Anxiety Symptoms	C	C			C	C
Children Depression Inventory-2 (CDI-2)	Depressive symptoms	C	C			C	C
The EuroQol 5D (EQ-5D)	Quality of life	P				P	P
The EuroQol 5D- Youth (EQ-5D-Y)	Quality of life child	C				C	C

		Every treatment session (12 assessments)					
Outcome Rating Scale (ORS)	Well-being	P					
Session Rating Scale (SRS)	Working alliance	P					
Global Clinical Impression Severity and Improvement Scale (CGI-S & CGI-I)	Illness severity and improvement	Tp					

*Moderators and predictors*							
Demographic variables	Demographics	P					
Structures Clinical Interview for DSM-5 Disorders in children and adolescents (SCID-5 Junior)^a^	Psychiatric comorbidity child	P/C				P/C	
Social Responsiveness Scale-2 (SRS-2)	Autism Symptoms	P					
Dutch Parenting Stress Questionnaire (OBVL)	Parental stress	P				P	P
Adult Self Report (ASR)	Emotional and behavioral problems parent	P				P	P

P: Parent(s); C: Child; T: Teacher; Tp: Therapist ^a^Through online interview.

### 2.6 Therapist training and treatment integrity

The therapists in our study are four experienced clinical therapists who received training (a 2-day workshop) from the developers of the SPACE treatment. The standardized Dutch treatment manual for therapists [35] and a workbook for parents is used in all treatment sessions. Protocol adherence is safeguarded via supervision once every other week by an experienced, certified SPACE supervisor. To ensure treatment integrity, all 12 treatment sessions will be video recorded. 20 % of the sessions will be randomly selected and rated on protocol adherence by two independent, trained research assistants. If participants do not give consent for video-recording during treatment, they can still participate in the study. After every treatment session, therapists complete a checklist of treatment components and report on what they discussed or practiced in the treatment session to enhance protocol adherence.

### 2.7 Assessments

*Frequent assessments.* The frequent assessments obtain the primary outcome measures described as the ‘frequent measure on OCD and FA’, used for SCED analyses. During all phases (A-B1-B2-C), parents and children receive four short questions three times per week (on Mondays, Wednesdays, and Fridays) on OCD severity and FA. This will result in 72 frequent assessments for each participant (i.e. 72 assessments for each parent and 72 assessments for the child) over the total course of the study. It will result in approximately 12–24 frequent assessments during the baseline phase, 36 frequent assessments during the total treatment phase and again 12-24 frequent assessments during the follow-up phase. Participants are also asked to report any special events during the past week, each Friday during all phases. The measures are collected electronically using the smartphone application m-Path [36], designed to facilitate frequent assessments over time of participants feelings, thought, behaviors an experiences as they occur in daily life. This research methodology is often referred to as experience sampling method (ESM) [37].

*Standard clinical assessments*. The standard clinical assessments at timepoint T0-T5 and during every treatment session obtain the secondary outcomes measures, moderators and predictors of treatment response. An overview of all measures is listed in Table 2.

### 2.8 Primary outcome measure

*Frequent measure on OCD and FA.* The frequent measure is a brief 4-item questionnaire on the level and time spent on OCD and FA (see Table 3). The questions are developed in collaboration with experience experts from patient organizations and families who previously received therapy for their child's OCD. Each item is rated on a 11-point scale (0–10). Both parents and the child complete the questions independently of each other.

**Table 3:** Frequent brief assessment questions on OCD and FA for parents and children.

	Question	Scale
OCD questions parents		
1	To what extent did your child experienced obsessions and compulsions today (including the night)?	0 (*not at all*) to 10 (*to the fullest extent*)
2	How much time did your child spend on obsessions and compulsions today (including the night)?	*0 min* to *more than 8 h*
FA questions parents		
3	To what extent did you accommodate to the obsessions and compulsions of your child today (including the night)?	0 (*not at all*) to 10 (*to the fullest extent*)
4	How much time did you spent accommodating to the obsessions and compulsions of your child today (including the night)?	*0 min* to *more than 8 h*
OCD questions child		
1	To what extent did you experienced obsessions and compulsions today (including the night)?	0 (*not at all*) to 10 (*to the fullest extent*)
2	How much time did you spend on obsessions and compulsions today (including the night)?	*0 min* to *more than 8 h*
FA questions child		
3	To what extent did your parents accommodate to your obsessions and compulsions today (including the night)?	0 (*not at all*) to 10 (*to the fullest extent*)
4	How much time did your parents spent accommodating to your obsessions and compulsions today (including the night)?	*0 min* to *more than 8 h*

### 2.9 Secondary outcome measures

*Children's Yale-Brown Obsessive-Compulsive Scale*. OCD symptoms and OCD severity will be assessed with the Children's Yale-Brown Obsessive-Compulsive Scale (CY-BOCS) [31], a clinician rated semi-structured interview based on a combined parent and child-report and clinical evaluation. The CY-BOCS consists of a symptom checklist and a severity scale. The symptom checklist assesses current and past obsessive and compulsive experiences (indicated with ‘yes’ or ‘no’). The severity scale consists of 5 items on obsession severity and 5 items on compulsion severity, rated on a scale from 0 to 4. The total score is the sum of the obsessive and compulsive scale (0–40). A higher score indicates more severe OCD symptomatology. The CY-BOCS has good reliability (*α* = 0.87) and construct validity [38]. Comparable properties have been found for the Dutch translation of the CY-BOCS [39].

*Family Accommodation Scale-Anxiety.* The Family Accommodation Scale – Anxiety (FASA) [11] is a parent report measure and comprises 9 items rated on a Likert scale from 0 (never) to 4 (daily) that add up to the total accommodation score. Four additional items, rated from 0 (no) to 4 (extreme), assess the distress and consequences of family accommodation. The FASA-CR is a child-rated version with comparable items. The FASA (*α* = 0.87) and FASA-CR (*α* = 0.79) have shown good test-retest reliability in English speaking samples [40]. The validation of the Dutch version of the FASA and FASA-CR is currently in progress in a parallel study.

The *Coercive Disruptive Behavior Scale* – *Pediatric OCD* (CD-POC) [19,41] will measure coercive and disruptive behaviors of the child, that may lead to increased family accommodation [41]. Validation of the Dutch translation of the CD-POC is currently in progress. Emotional and behavioral problems of the child are assessed with the *Child Behavior Checklist* (CBCL, parent-report)*,* and its parallel versions: the *Teacher Report Form* (TRF; teacher-report) and the *Youth Self Report* (YSR, child-self-report, for children older than 11 years old) [42,43]. The *Screen for Childhood Anxiety Related Emotional Disorders-NL* (SCARED-NL, child-report) [44] is used to measure symptoms of anxiety. Symptoms of child depression are assessed by the *Children Depression Inventory-2* (CDI-2, child-report) [45,46]. Quality of life is measured using the *EuroQol 5D* (EQ-5D) for parents and *EuroQol 5D-Youth* (EQ-5D-Y) for children [47]. All instruments demonstrated sufficient to good psychometric qualities [19,42,45,[48], [49], [50]].

The *Clinical Global Impression-Severity and -Improvement scale* (CGI-S & CGI-I) [51,52] is used to measure change in psychopathological problems over time and filled in after every treatment session by the therapist. The CGI is found to be a reliable and valid instrument for routine use to measure clinical change [48]. The *Outcome Rating Scale* (ORS) and *Session Rating Scale* (SRS) [53,54] reported by parents is used to assess the treatment process. The ORS is filled in before the next session to report about past week and the SRS after every treatment session to report on the working alliance between parents and therapist. Higher scores indicate a higher well-being and treatment satisfaction. The ORS and SRS have shown good reliability and stable concurrent and construct validity [53,54] In addition, treatment satisfaction is evaluated directly after the end of the treatment (T4) with a 11-item questionnaire filled in by parents.

### 2.10 Moderators and predictors of treatment outcome

Demographic variables such as gender, age of child, cultural background, family functioning, educational level, school functioning and life-events are assessed during intake and by an online standardized questionnaire. Psychiatric co-morbidities of the child will be assessed by the *Structured Clinical Interview for DSM-5 disorders* (SCID-5 junior) a reliable and valid instrument to assess DSM-5 diagnosis in children and adolescents [32]. Symptoms of autism reported by parents are assessed using the *Social Responsiveness Scale* (SRS-2) [55,56]. Reliability and criterium validity statistics are good [57].

Parental burden is measured with the Dutch *Parenting Stress Questionnaire* (Opvoedbelasting Vragenlijst; OBVL) [58], a self-report questionnaire that addresses five aspects: problems in the parent-child relationship, problems in parenting, depressed moods, role restriction, and health complaints. The Dutch Committee on Test and Testing rated the reliability of the OBVL as good and the constructed validity as sufficient [58]. The *Adult Self Report* (ASR) [59] is used to assess parental behavioral and emotional problems. The ARS is a reliable and valid instrument to measure adult psychopathology [60].

## 3 Statistical analysis

### 3.1 Plan of statistical analysis for the SCED primary study parameters

In this study, the main objective is to explore the efficacy of SPACE. The primary analysis focuses on the change in the frequent measure on OCD and FA between baseline phase (A) and treatment phase (B1+B2). First, individual participants data will be separately systematically visually analyzed [61]. Next, individual data will and modelled using a generalized least squares (GLS) approach (assuming autocorrelation) [62]. To test the treatment effect on group level, a multilevel modelling approach [63] based on the examples of Shadish and colleagues (2013) [64] and Moeyaert and colleagues [63,65,66] will be used. Data from all individual participants will be included in the multilevel model and structured across a hierarchical three-level structure. Level 1 represents the frequent measurement occasions, with measures being nested within each participant. Level 2 represents the individual participants (mothers, fathers and children), with family members being nested in a family unit. Level 3 represents the family units (e.g. members of one family) included in this study. Adding time-variant predictors (the number of measurement occasion to model general time trend, a dummy-coded variable (0–1) representing the phase and an interaction term between these two) at Level 1 allows to explore for a shift in level of OCD and FA at the start of the treatment phase and the shift in trend during the treatment phase [65]. Multiple plausible multilevel models will be estimated to enhance conclusions about the treatment effect [63]. For these analyses a significance level of 0.05 is used.

Exploratory, we expect the greatest shift in OCD severity and FA level in phase B2 when parents implement their plan to reduce FA. By starting phase B2 directly after implementation of the plan we aim to explore the immediate effect of the implementation and shift in OCD and FA level during the second half of the treatment. For this purpose, an additional time predictor at Level 1 will be added to estimate shift in level and shift in trend for phase B2.

For the secondary objectives, cross-level interactions will be added to the multilevel modelling approach to explore moderators effecting the efficacy of SPACE. To explore mechanisms of change, cross-lagged correlations will be calculated to explore the association between the frequent measure of OCD and FA over time (with respect to the direction of the association as well as the time-dependent association; [23,25]. Recent developments in the field of single-case mediation analysis (SCMA, [67]) will be used to explore mediation pathways.

### 3.2 Plan of statistical analysis for the secondary study parameters

Considering the standard clinical measurements on OCD severity, FA and secondary outcomes measures, reliable change indices (RCIs) [68] will be calculated for each individual participant to detect clinical relevant changes between all six assessment occasions (T0-T1-T2-T3-T4-T5), with the comparison between baseline (T0) and post-treatment (T4) as most relevant. RCI's will be calculated separately for each informant to collect meaningful information on differences between informants within one family. The RCI is a variable with three possible values: no reliable change, reliable deterioration and reliable improvement.

## 4 Discussion

Pediatric OCD is a severely impairing disorder, and additional alternative treatment options are needed, especially for the 40 % of those children that did not benefit from or refuse first-line treatments. To our knowledge, this study is the first to explore the innovative parent-based SPACE treatment in youth with OCD using a SCED design. The strength of this methodology is that, while targeting family accommodation, detailed insight into longitudinal changes in parent-child dynamics and the longitudinal relation between OCD severity and FA over time will be obtained. The study also aims to contribute to knowledge about targeting FA in youth with OCD, by using multiple informants (parents, child, teacher, clinicians) and including secondary outcomes and predictors of treatment success.

### 4.1 Strengths and limitations

This study has some limitations that need to be addressed. First, our aim to include children who either did not benefit from previous treatments or were unable to adhere to individual treatment protocols results in a heterogeneous sample of children with severe or complex OCD symptomatology. This heterogeneity arises from including children who, despite having treatment motivation, did not respond to previous treatments, as well as those children with low motivation for individual treatment due to low insight and low motivation to change their OCD symptoms. Low treatment motivation among these children could also result in low motivation to participate in this study, complicating data collection efforts, leading to potential biased results. Including this group of complex children, however, reflects the challenging clinical practice, strengthens the ecological validity and representativeness of our findings, and strengthens the cross-cultural evidence for parent-based treatments for youth with OCD. Second, during this study children are not participating in individual psychotherapy, in order to assess the efficacy of SPACE as a stand-alone treatment option. This limits the generalizability of the findings of SPACE as an additional treatment on top of individual treatment for the child. However, determining the optimal place of SPACE within the set of treatment options for youth with OCD is considered a future research purpose. At last, at the start of this study the validated Dutch version of the FASA, FASA-CR and CD-POC was not yet available. A validation study is conducted simultaneously with this study.

### 4.2 Implications for clinical practice

The results of this study can have great implications for clinical practice considering the high need for additional and alternative treatment options for children with OCD who did not benefit from first line treatment options. Providing cross-cultural evidence that an innovative parent-based treatment is effective in reducing OCD and FA in complex cases and unravel underlying mechanisms of treatment success, will contribute to improvement of clinical care. Then SPACE can be provided as an additional or alternative treatment for youth with OCD. Proven effective, there is potential and a great interest from clinical practice for dissemination of SPACE in our country.

## CRediT authorship contribution statement

**Julia D.K. Veeger:** Writing – original draft, Resources, Project administration, Methodology, Investigation, Conceptualization. **Luuk Stapersma:** Writing – review & editing, Supervision, Methodology, Funding acquisition, Conceptualization. **Eli R. Lebowitz:** Writing – review & editing, Conceptualization. **Bonne Zijlstra:** Writing – review & editing, Methodology, Conceptualization. **Ramón Lindauer:** Writing – review & editing, Supervision, Conceptualization. **Elisabeth M.W.J. Utens:** Writing – review & editing, Supervision, Funding acquisition, Conceptualization. **Chaim Huijser:** Writing – review & editing, Supervision, Methodology, Funding acquisition, Conceptualization.

## Available data and materials

Not applicable. This paper presents the study protocol and does not contain any data or results.

As of October 2024, 9 families have been enrolled in the study at the time of submission. Data collection is projected to be completed in 2025.

## Trials registration

ClinicalTrials.gov Identifier: NCT06356090.

## Ethical approval and consent to participate

The Medical Ethics Committee of the Amsterdam University Medical Centers has approved this trial. (METC number: NL84369.018.23). The study will be conducted according to the Helsinki Declaration and its later amendments or comparable ethical standards. Informed consents will be obtained for the parents or guardians of the participating families, and from children (12+) themselves. This article does not contain any studies with animals performed by any of the authors.

## Funding

This work was supported by Fonds Stichting Gezondheidszorg Spaarneland (Grand ID: 2022415). The funding source had no role in the design of the study, and will not have any role in its execution, analysis, interpretation of the data, or decision to submit results.

## Declaration of competing interest

The authors declare the following financial interests/personal relationships which may be considered as potential competing interests: Dr. C. Huijser reports financial support was provided by Fonds Stichting Gezondheidszorg Spaarneland. Dr. Lebowitz receives royalties from books (including on SPACE, the treatment examined in this study) and revenue from clinical training workshops. If there are other authors, they declare that they have no known competing financial interests or personal relationships that could have appeared to influence the work reported in this paper.
